# Experimental yellow fever virus infection in the squirrel monkey (*Saimiri spp*.) I: gross anatomical and histopathological findings in organs at necropsy

**DOI:** 10.1590/0074-02760190501

**Published:** 2020-11-09

**Authors:** Milene Silveira Ferreira, Pedro Soares Bezerra Júnior, Valíria Duarte Cerqueira, Gabriela Riet Correa Rivero, Carlos Alberto Oliveira Júnior, Paulo Henrique Gomes Castro, Gilmara Abreu da Silva, Wellington Bandeira da Silva, Aline Amaral Imbeloni, Jorge Rodrigues Sousa, Ana Paula Sousa Araújo, Franko de Arruda e Silva, Robert B Tesh, Juarez Antônio Simões Quaresma, Pedro Fernando da Costa Vasconcelos

**Affiliations:** 1 Instituto Evandro Chagas, Seção de Arbovirologia e Febres Hemorrágicas, Ananindeua, PA, Brasil; 2 Universidade Federal do Pará, Programa de Pós-Graduação em Biologia de Agentes Infecciosos e Parasitários, Belém, PA, Brasil; 3 Universidade Federal do Pará, Instituto de Medicina Veterinária, Laboratório de Patologia Animal, Castanhal, PA, Brasil; 4 Instituto Evandro Chagas, Centro Nacional de Primatas, Ananindeua, PA, Brasil; 5 University of Texas Medical Branch, Department of Pathology, Galveston, TX, USA; 6 Universidade do Estado do Pará, Departamento de Patologia, Belém, PA, Brasil

**Keywords:** yellow fever, Saimiri, pathophysiology, experimental infection

## Abstract

**BACKGROUND:**

Non-human primates contribute to the spread of the yellow fever virus (YFV) and the establishment of transmission cycles in endemic areas.

**OBJECTIVE:**

To describe the severe histopathological aspects of YFV infection, 10 squirrel monkeys were infected with YFV and blood, brain, liver, kidney, spleen, heart, lung, lymph node and stomach were collected at 1-7, 10, 20 and 30 days post-infection (dpi).

**METHODS:**

Histopathological analysis and detection of the genome and viral antigens and neutralising antibodies were performed by RT-PCR, immunohistochemistry and neutralisation test, respectively.

**FINDINGS:**

Only one animal died from the experimental infection. The genome and viral antigens were detected in all investigated organs (1-30 dpi) and the neutralising antibodies from seven to 30 dpi. The brain contained perivascular haemorrhage (6 dpi); in the liver, midzonal haemorrhage and lytic necrosis (6 dpi) were observed. The kidney had bleeding in the Bowman’s capsule and tubular necrosis (6 dpi). Pyknotic lymphocytes were observed in the spleen (1-20 dpi), the lung had haemorrhage (2-6 dpi), in the endocardium it contained nuclear pyknosis and necrosis (2-3 dpi) and the stomach contained blood in the lumen (6 dpi).

**MAIN FINDINGS:**

Squirrel monkeys reliably reproduced the responses observed in human cases of yellow fever and, therefore, constitute an excellent experimental model for studies on the pathophysiology of the disease.

Yellow fever virus (YFV) is member of the family *Flaviviridae*, genus *Flavivirus.* YFV has single-stranded RNA genome, positive polarity and only one serotype.^1^ Some non-human primates (NHP) are excellent sentinels because their deaths following natural infection with YFV are an indicator of virus activity, which alerts public health agencies to begin efforts to prevent the virus spreading into urban areas.^2^ Although urban yellow fever (YF) has not been registered in Brazil since 1942, the sylvatic cycle manifests itself in an irregular way, with sporadic cases in forested areas (Amazon) during interepidemic periods. The recent extensive YF epidemic in Brazil (2017-2018) has brought to the fore the possibility of re-emergence of the virus in urban areas and the need for the organisation of public health services to control and combat the disease.^3^

The susceptibility of NHP to YFV was originally demonstrated in 1927 with the viral prototype (Asibi strain) in rhesus monkeys^4^ ^)^ and subsequently with descriptions of experiments with the capuchin (*Cebus*) monkey (*Sapajus*), woolly monkey (*Lagothrix*), spider monkey (*Ateles*), squirrel monkey (*Saimiri*) and marmosets (*Callithrix*).^5^ ^,^ ^6^ The infection presents differently among NHP; *Macaca mulatta* (rhesus monkeys) and monkeys the genera *Alouatta*, *Ateles*, *Saimiri* and *Callithrix* are more susceptible to YFV compared to *Sapajus*, that are easily infected but have a low lethality rate.^2^

Most information about the pathology of YFV infection is derived from experimental studies of Old-World monkeys and from fatal human cases.^7^ ^,^ ^8^ These descriptions emphasise the intense hepatic damage and the selectivity of the virus for the midzonal region of the liver. In this context, studies on the pathophysiology with systematised descriptions of the histopathological changes of YF in New World NHP models are still little explored in the literature. Monkeys of the genus *Saimiri* are widely distributed throughout the Amazon,^9^ indicating a potential role as YFV primary hosts, viral amplifiers and sources of information on the dynamics of virus circulation during the between epidemic period and epidemics.

In view of the above, we examined the pathological aspects of YFV infection in experimentally infected squirrel monkeys (*Saimiri* spp.) in order to better understand the pathophysiology of the disease, to contribute to the establishment of diagnostic criteria for the disease, as well as to stimulate future studies on critical events in the pathogenesis of the disease, organ-specific viral therapies and vaccine-induced adverse events.

## MATERIALS AND METHODS

*Virus -* A strain of the South American genotype I (BeH655417) was used, originally isolated from a serious human case in Roraima in the year 2014 available by the Evandro Chagas Institute (IEC) and a single mouse passage. Viral propagation was performed by additional passage in a continuous cell line of *Aedes albopictus* (clone C6/36)^10^ and confirmation of cell infection was performed using monoclonal antibodies (Bio-Manguinhos/Fiocruz, Brazilian Ministry of Health, *Health Surveillance*Secretariat) by the indirect immunofluorescence test.^11^

*Animals -* Eleven male squirrel monkeys (*Saimiri* spp.), males, adults (six to seven years old) with an average weight of 786 g from a breeding colony at the National Primate Centre (CENP) were used in this study. All animals were negative for anti-flavivirus antibodies (YFV, dengue virus 1-4, Ilheus virus, Rocio virus, Sant Louis virus) in the haemagglutination inhibition test.^12^ ^,^ ^13^ The animals were distributed in individual stainless steel cages with retractable bottom (0.80 m long × 0.80 m wide x 0.90 high) and were fed with PNH specific Megazoo P25 ration, mix of fruits, vegetables, tubers, milk and eggs and water at will, according to the nutritional protocol recommended by CENP. All procedures were performed at biosafety level 3 at the IEC. 

**Fig. 1:**
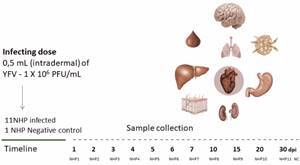
experimental kinetics of the study with star monkey infected with the virus of the experimental kinetics of the study with star monkey infected with the yellow fever virus (YFV). Non-human primates (NHP) (*Saimiri* spp.) were infected with 0.5 mL of the yellow fever virus intradermally infective dose = 1 x 10^6^ plaque forming units (PFU)/mL and one animal was kept until the end of the experiment as a negative control (NC). One animal was sacrificed on pre-established days post-infection (dpi) for the collection of blood, brain, lymph node, heart, lung, liver, stomach and kidney samples.

Ten of the animals were infected with the BeH655417 isolate [infectious dose: 1 × 10^6^ plaque forming units (PFU)/mL] via the intradermal route in the third right intercostal space. The 11th animal was maintained as a negative control until the end of the experiment. In the first seven days and at 10, 20 and 30 dpi, an animal received prior anaesthesia, ketamine hydrochloride (10-15 mg /kg) and xylazine hydrochloride (1 mg/kg) by intramuscular route. The overdose was performed with fentanyl (40 mcg/kg) and ketamine hydrochloride (20 mg/kg) intravenously^14^ (Fig. 1). To examine the carcass, mucous membranes and organs of the animals, a form was used with the parameters and scores that characterised the findings and the changes were recorded with the Fujifilm HS10 digital camera. Blood and fragments of the brain, liver, kidney, spleen, lymph node, lung, heart and stomach were collected in triplicate, two samples were stored at -70^0^C and one in 10% neutral formalin buffer until processing.

*Viral load and serology -* Total RNA was extracted with the Maxwell 16 Viral Total Nucleic Acid Purification kit (Promega, Madison, USA) and the Maxwell extraction platform (Promega). The viral load was quantified according to.^15^ For this purpose, the Go Taq Probe 1-Step RT-qPCR System commercial kit (Promega) was used in conjunction with the absolute quantification method using a pGEM Easy (Promega) vector cloned plasmid from the YFV genome. The GoTaq qPCR Master Mix (Promega) kit, processed in the ABI Prism 7500 Sequence Detection System (Applied Biosystems, Foster, USA) and the YFV F-GCTAATTGAGGTGYATTGGTCTGC primers were used for real-time quantitative (RT-qPCR) and YFV R-CTGCTAATCGCTCAAMGAACG. The RT reaction occurred in cycles of 50^0^C for 5 min and 95^0^C for 2 min, followed by 45 cycles of 95^0^C for 3 s, 60^0^C for 30 s and finally 4^0^C to infinity. Viral load was expressed as number of viral particles/μL. For the detection of antibodies, the mouse neutralisation test (MNT) was performed.^16^ For this, the lethal dose (LD)_50_ (per 0.02 mL)^17^ was calculated and samples with a logarithmic neutralisation index (LNI) greater than or equal to 1.7 were considered positive.

*Histopathology and immunohistochemistry (IHC) -* Tissue samples were fixed in 10% neutral buffered formalin, routinely processed, embedded in paraffin, sectioned at 5 μm and stained with haematoxylin and eosin (*H&E*) for histological examination. The detection of YFV antigens in tissues by IHC was based on the formation of a biotin complex with streptavidin alkaline phosphatase.^18^ The VECTASTAIN^®^ABC Kit was used, according to the manufacturer (Thermo Scientific, EUA): primary mouse monoclonal anti-VFA antibody, produced at IEC,^18^ diluted 1:200 in PBS; secondary anti-mouse biotinylated horse IgG antibody diluted 1: 5 in PBS. The primary-secondary antibody complex was linked to streptavidin with various biotin molecules conjugated to the alkaline phosphatase enzyme at 1:20 dilution in PBS, followed by the substrate. The slides were stained with haematoxylin and fixed with entelan^®^. Positive and negative NHP controls were included.

Tissue specimens were analysed by optical microscope (AXIO IMAGER Z1-ZEISS-model 4560006, Carl Zeiss, Germany). For the semiquantitative analysis, a 1 cm^2^ graduated reticulum (area 0.0625 mm^2^) was used under a 400X objective. Ten microscopic fields were counted and the mean of the immunolabelled cells was calculated for each area investigated and the result was divided by 0.0625 mm^2^.^8^

*Ethics -* Was approved by the Ethics Committee on the use of Animals (protocol 0014/2014) and [National System of Biodiversity Information (Sisbio)/Chico Mendes Institute for Biodiversity Conservation (ICMBio)] (protocol: 38744-1). 

## RESULTS

**Fig. 2:**
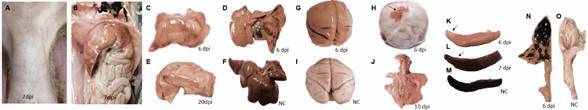
gross photographs changes observed in squirrel monkeys (*Saimiri* spp.) infected by the yellow fever virus (YFV). (A) Ventral back of the abdomen with bleeding points at 7 days post-infection (dpi). (B) Diffuse yellow liver with haemorrhagic spots and subcapsular multifocal petechiae at 7 dpi. (C, D) Heavy bleeding throughout the liver of the animal that succumbed to 6 dpi. (E) Liver diffusely yellowed at 20 dpi. (F) Normal liver. (G) Brain with congestion and haemorrhage. (H) Skull lid with subcutaneous hematoma at 6 dpi (arrow). (I) Normal brain. (J) Marked pulmonary congestion (left). (K, L) Spleen with rounded edges and whitish and/or dark red areas (arrows). (M) Normal spleen. (N) Stomach with contents (blood/digested) in the lumen. (O) Normal stomach. NC: negative control.

The study lasted for a period of 30 days. Bleeding points on the skin were seen on the ventral back at 7 dpi (Fig. 2A). In the liver, a marked follicular pattern (more reddish areas interspersed with paler areas) was observed at 4-5 dpi with subcapsular multifocal petechiae (7 dpi) (Fig. 2B), severe bleeding (6 dpi) (Fig. 2C, D) and diffuse jaundice (2-20 dpi) (Fig. 2E). Axillary lymph nodes located near the inoculation area presented with infarcts (1-2 dpi, data not shown). The brain exhibited moderate congestion in the leptomeninges with perivascular haemorrhages (1-6 dpi) and a hematoma in the skullcap (6 dpi) (Fig. 2G, H). The right lung was slightly reddish homogeneously (10 dpi) (Fig. 2J). The spleen borders were rounded (5-20 dpi) and white or dark red areas were visualised on the capsular surface (6-7 dpi) (Fig. 2K, L). The heart was pale between 4 and 7 dpi (data not shown), and the stomach showed moderate bleeding in the lumen (6 dpi) (Fig. 2N). One animal died at 6 dpi presumably due to severe disease. No macroscopic changes were observed in the organs of the animal that survived up to 30 dpi, suggesting the complete regeneration of the same. Negative controls of the liver, brain, spleen and stomach are represented in Figs. 2F, 2I, 2M and 2O and showed no changes.

**Fig. 3:**
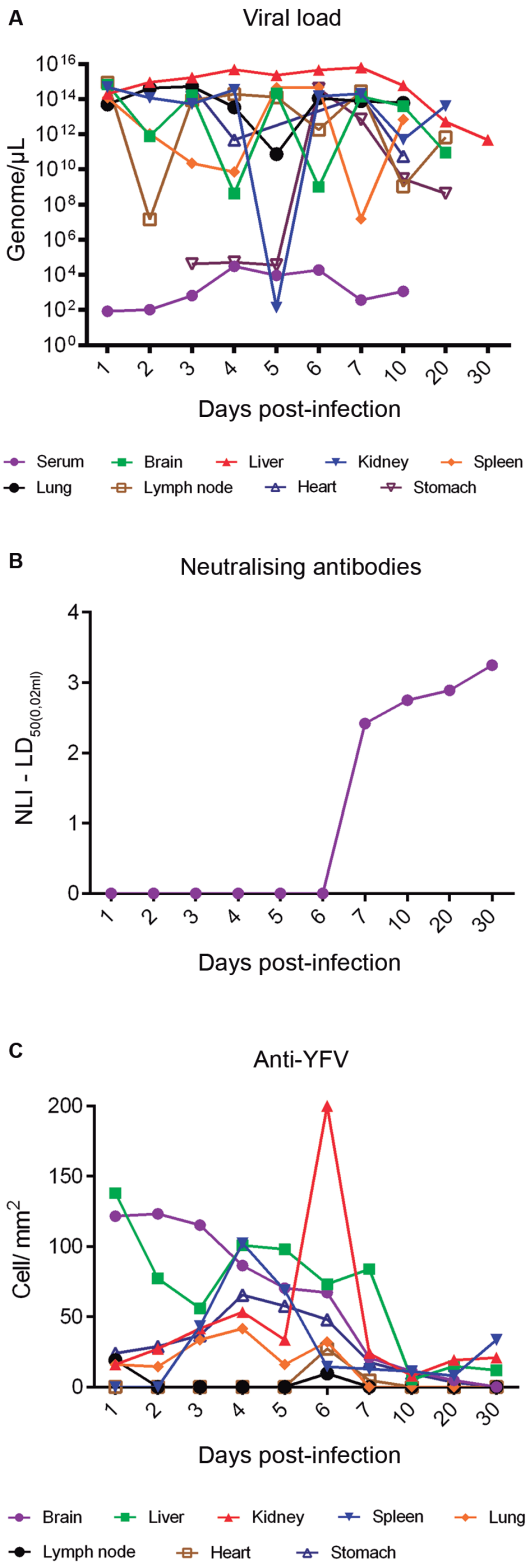
detection of the genome, viral antigens and neutralising antibodies in monkeys’ squirrels (*Saimiri* spp.) infected with yellow fever virus (YFV). (A) Detection of viral RNA by RT-qPCR. (B) Detection of neutralising anti-YFV antibodies. (C) Detection of viral antigens by immunohistochemistry. LD_50_: lethal dose; LNI: logarithmic neutralisation index.

The viral genome was detected irregularly in all experimentally infected organs. In the brain, kidney, lymph node and stomach viral RNA was detected between 1-20 dpi, in the spleen, lung and heart between 1-10 dpi and in the liver between 2-6 dpi. Viral load peaks were observed at 1 dpi in the lymph nodes, brain and kidney, 3 dpi in the lung and heart, 4 dpi in the liver and 6 dpi in the spleen and stomach (Fig. 3A). Neutralising antibodies were detected between 7-30 dpi by MNT (Fig. 3B).

**Fig. 4:**
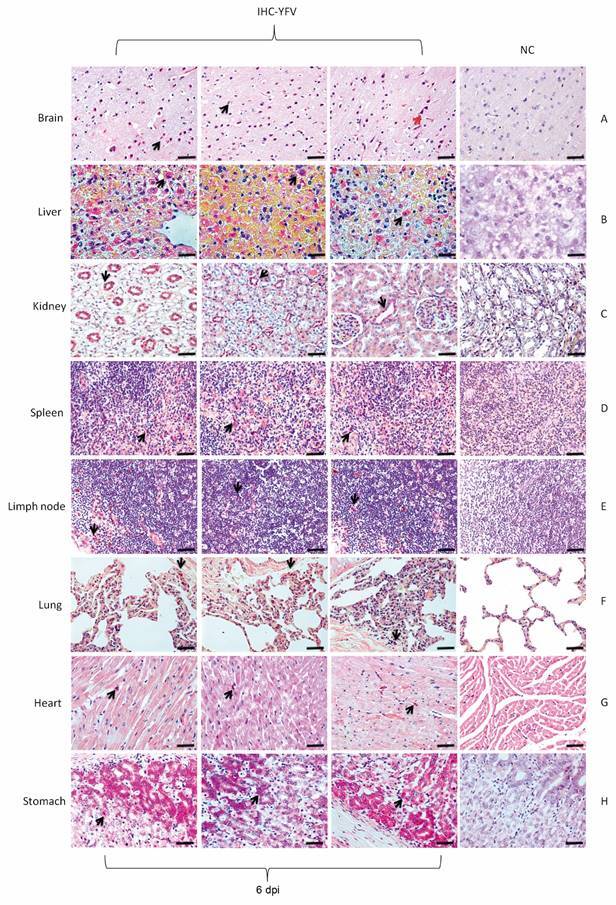
detection of viral antigens in the organs of the squirrel monkeys (*Saimiri* spp.) infected with yellow fever virus (YFV) that died at 6 days post-infection (dpi). (A) In the brain, immunostaining was predominant in glial cells in the neural parenchyma (black arrow) and neurons (red arrow). (B) YFV tropism by infecting hepatocytes (black arrow) was obvious at 6 dpi. (C) In the kidney, the immunostaining was marked in the renal tubules (black arrow). (D, F) In the spleen, lymph node and lung, immunostaining was enhanced in the mononuclear cells. (G, H) Myocardial and stomach cells were the preferred target of YFV at 6 dpi (black arrow). 400X (20 µm). IHC: immunohistochemistry; NC: negative control.

Viral antigens were detected by IHC in all organs of the infected monkeys. In the brain, liver, kidney, spleen and stomach, YFV antigens were detected between 1-30 dpi. In the brain, kidney, lung and stomach, increased immunostaining was visualised at 1-6 dpi. In the lymph node, lung and heart, immunostaining was irregular and was observed up to 6 dpi (Fig. 3C). Viral antigens were detected in all organs of the animal that died (6 dpi) and the kidney was the organ with the highest number of immunolabelled cells (268.80 cells/mm^2^). Fig. 4 highlights the immunostaining in organs of the animal that died 6 dpi.

**Fig. 5:**
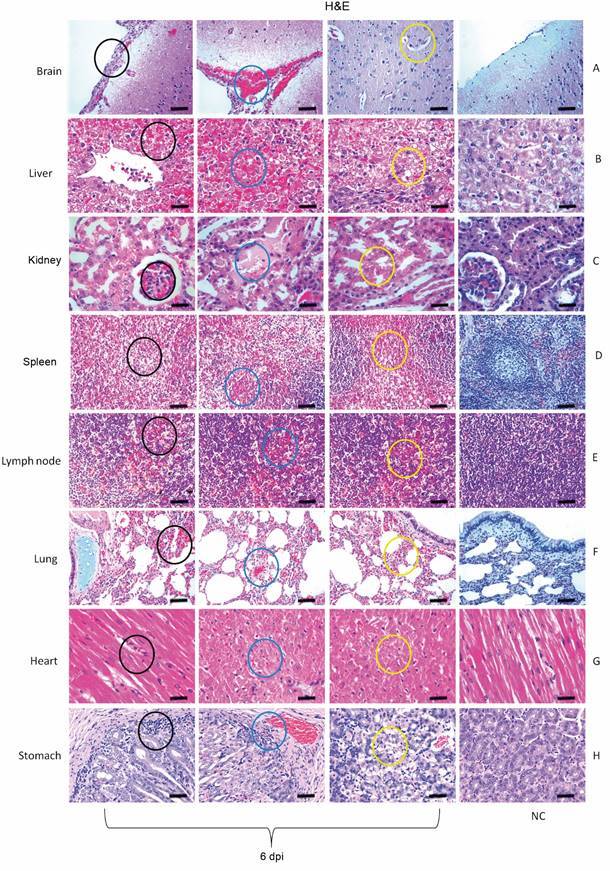
anatomopathological changes observed in the organs of the squirrel monkeys (*Saimiri* spp.) infected with yellow fever virus (YFV), which died at 6 days post-infection (dpi). (A) Thickening of the meninges (black circle) with oedema, congestion and haemorrhage blue circle. Oedema in the neural parenchyma was characteristic (yellow circle). (B) In the liver, tissue damage was characterised by centrilobular and midzonal haemorrhage (black circle) and lytic necrosis (blue and yellow circle). (C) In the kidney, there were haemorrhagic points in Bowman’s capsule (black circle) and tubular necrosis was a relevant finding (blue and yellow circle). (D) The deleterious effects on tissue in the spleen are manifested mainly in haemorrhagic foci (black, blue and yellow circle). (E) In the lymph node, bleeding was evidenced in the centre of the cortical region (black, yellow and blue circle). (F) Pulmonary parenchyma showed congestion and haemorrhage (black, blue and yellow circle). (G) In the heart, nuclear pyknosis in myocardial cells was impressive (black, blue and yellow circle). (H) In the stomach with inflammatory infiltrate (6 dpi) (black circle) and moderate numbers of acidophilic cells with nuclear pyknosis (blue and yellow circle). H&E: 400X (20 μm). NC: negative control.

In the dermis of the inoculated area, a mild multifocal perivascular mononuclear infiltrate was observed that was maintained throughout the experiment in all animals (1-30 dpi, data not shown). The animal that died (6 dpi) showed thickening of the meninges, oedema, congestion, and haemorrhage (Fig. 5A). The deep layers at white matter in the occipital cortex (6 dpi) exhibited areas of vacuolation of neuropilus where there are astrocytes with abundant eosinophilic cytoplasm; the latter were also observed occasionally in other areas in the white matter without vacuolisation. The cerebellum of a euthanised animal at 7 dpi also showed a discrete vacuolisation in the white matter. Hepatic damage of the infected animals was marked by centrilobular and midzonal haemorrhage, steatosis and lytic necrosis mainly of hepatocytes (1-6 dpi) (Fig. 5B). In the kidney, an infiltrate with light multifocal mononucleated was seen (2-6 dpi). Light diffuse congestion occurred in the lung (5 and 10 dpi), with rare areas of tubular mineralisation in the spinal cord (10-20 dpi). In the animal that died (6 dpi), tubule cell cytoplasm vacuolisation, haemorrhagic spots on Bowman’s capsule and tubular necrosis were observed in the kidney (Fig. 5C). The spleen showed rare pyknotic lymphocytes and nuclear debris (1-20 dpi) with white and red pulp hyperplasia and haemorrhagic foci (6 dpi) (Fig. 5D).

The axillary lymph nodes near the inoculated area had moderate haemosiderosis, but no evident follicles (2-6 dpi). Splenic follicles showed slight rarefaction with pyknotic nuclei (3 dpi), prominent cortical interspersed by tumefied cell nuclei, prominent nucleoli and vacuolated cytoplasm (macrophages or large lymphocytes) (4 dpi). Bleeding was also observed in the centre of the cortical region (6 dpi) (Fig. 5E). There was mild diffuse lymphoid hyperplasia and congestion (7 dpi) with necrosis in the follicles that was characterised by the presence of debris in the macrophage cytoplasm. There was a small amount of foamy macrophages in the spinal cord and occasional light vacuoles between the lymphocytes in the cortical region (10-30 dpi). In the lung, oedema, congestion, severe haemorrhage and mononuclear inflammatory infiltrate (6 dpi) (Fig. 5F) and mild diffuse congestion was seen 1-30 dpi. The myocardium in areas close to the endocardium contained myofibres with increased eosinophilia, some with nuclear pyknosis and cytoplasmic vacuoles (1 and 6 dpi) (Fig. 5G). Discrete areas of incipient ischemic necrosis in the endocardium, bordered by vacuolated myocyte, were observed at 2-3 dpi. In the stomach (Fig. 5H), inflammatory infiltrate (6 dpi), moderate numbers of acidophilic cells with nuclear pyknosis and increased eosinophilia (1-10 dpi) were observed. The detailed description of histopathological changes is described in the Supplementary data, Table.

## DISCUSSION

YF in NHP is an acute infection of variable severity. In rhesus monkeys and members of the *Alouatta* genus, the disease is fulminant due to the high susceptibility of these animals to the virus.^19^ Because of the abrupt onset and rapid course of infection, most NHPs show few signs of disease. Likewise, the clinical presentation of infection in humans ranges from asymptomatic to severe and sometimes fatal haemorrhagic disease.^20^ The difficulty of accessing the NHP restricted the use of one animal per day during experimental kinetics. However, this did not limit the study, as we observed that most animals developed clinical signs commonly exhibited by humans and we presume complete organ recovery, as seen at 30 dpi. YFV infection in squirrel monkeys was viscerotropic and neutralising anti-YFV antibodies were detected in most animals (7-30 dpi). The animal that died from the experimental infection at 6 dpi showed severe histopathological changes in all organs.

After reaching the bloodstream, the YFV spread throughout the body and reached virtually every organ of the infected squirrel monkeys. This has been shown by detecting the viral genome in blood/serum (1-10 dpi) as well as brain, liver, kidney, spleen, lymph node, heart, lung and stomach fragments between 1-20 dpi and by the immunomarcation of YF viral antigens between 1-30 dpi in these organs. A peculiar characteristic evidenced in our study was the low viral load detected in the serum (10^5^ copies/μL) when compared to the other organs, in which 10^15^ copies/μL were detected on average (minimum value: 10^2^ copies/μL and maximum value: 10^15^ copies/μL). Low viral levels in the blood/serum reflect different phases of blood viral load dynamics, with viral compartmentalisation observed in several organs and the action of immune factors in the blood with the production of anti-YFV antibodies similar to that observed in HIV infection.^21^ Additional studies are needed to confirm such findings.

Haemorrhage was the most frequently observed change, but jaundice did not reach the intensity described in humans. Signs of bleeding of recent origin were visualised in the integument, liver, kidneys, lung, lymph node, stomach and brain. Petechiae visualised on the surface of slightly greenish skin (6-7 dpi) were smaller than those commonly found in humans.^22^ The histopathological findings in the skin showed the presence of a mononuclear inflammatory infiltrate around the site of virus inoculation. This probably represented the first stage of immune response to the virus, from which a response cascade will be triggered that will probably influence the clinical evolution of the infection as observed in other flaviviruses.^23^ Human axillary lymph nodes occasionally exhibit congestion, pallor and a moderate increase in size, as evidenced in the axillary lymph nodes nearly the site of virus inoculation in the animals of in this study. This increase probably reflects the host’s immune response to viral inoculation into the skin. After being phagocytosed, cells presenting viral antigens migrate to the regional lymph nodes where they are processed and presented to naïve CD4+ T lymphocytes, with the initiation of the effector immune response cascade.^24^ Likewise, the hyperplasia of splenic white pulp, in addition to congestion, provides a histological picture of the spleen reaction to the infectious process.

Digested blood in the stomach occurs in almost all human YF cases. In NHP, it is evidenced at lower frequency, but is remarkably similar in appearance. In both cases, black vomit (haemolysed blood) is often associated with bleeding in the gastric mucosa and duodenum. YFV viral antigens were detected in the gastric mucosa, which had not previously been described in the literature.^5^ ^,^ ^6^ ^,^ ^20^ The presence of these antigens shows the direct viral tropism by the organ, which probably plays a role in the genesis of gastric haemorrhage described both in PNH and in humans**.** In addition, the presence of mild multifocal mononuclear inflammatory infiltrate, as well as the intense congestion, also point to a role of the gastric immune system in the compartmentalised immune response to the virus, and its probable influence on the vascular alterations of the gastric mucosa that contribute to gastric haemorrhage and are described in other sites or organs in the course of the disease.^25^

In the liver of the animal (6 dpi) that died of YFV infection, the main lesion observed was a necrotising hepatopathy, which extended from the centrilobular to the midzone region and many aspects resemble that observed in human cases of YF.^8^ It is important to point out that the descriptions of the occurrence of midzonal liver lesions is also associated with other infections, as well as non-infectious cases. Although marked in YF, it can no longer be considered pathognomonic of the disease. Furthermore, it is not present in all NHP species, such as the marmosets.^26^

Kidney function is profoundly affected and plays an important role in the death of humans with YF. The renal necrotic process is mainly represented by acute tubular necrosis, which is due to vascular and haemorrhagic disorders in severe YF cases. But it is usually not massive and tends to be discontinuous in a way that resembles the pathology of the liver. Necrosis was not a visible trait in the NHP kidneys studied, although focal haemorrhagic changes and tubular cell changes were observed in our sample. In human cases, patients recovering from infection, the extent of necrosis is small and cellular losses are repaired through the hypertrophy of surviving cells, accompanied by complete tubular regeneration resulting from direct multiplication of the epithelium.^27^

In the lung, we observed intense intra-alveolar oedema and haemorrhage, which in both experimental models of YF in NHPs and in humans is likely the immediate cause of death. Haemorrhagic pulmonary involvement was intense, and these changes reflect the consequence of vascular involvement with organ haemorrhage. It is evident that the lung is a site of great importance in evolution of the disease, since its structure, rich in vascularisation, is certainly compromised by the vascular changes induced by the virus and the inflammatory factors that lead to endothelial alteration during the course of the disease. 

The myocardium presented areas close to the endocardium containing myofibrers with increased eosinophilia, some with nuclear pyknosis and cytoplasmic vacuoles (1 and 6 dpi). Discrete areas of incipient necrosis in the endocardium bordered by vacuolated myocytes were displayed at 2-3 dpi. Myocardial changes such as cytoplasmic eosinophilia, nuclear pyknosis and vacuolisation are characteristic of ischemic necrosis of the tissue and may occur due to vascular changes observed in severe cases of the disease. These changes may lead to functional alterations of the heart that, together with the pulmonary changes, contribute to death in these haemorrhagic cases.^27^

One of the surprising clinical aspects of YF in humans is the mental clarity of patients during the disease and close to death. In view of this, brain and central nervous system (CNS) injuries in humans are generally minimal.^27^ Among the rare reports on CNS involvement in humans with YF, we have macrophages containing blood pigment and cell infiltration in leptomeninges and chromatolysis of nerve cells and glial proliferation^28^ and small perivascular haemorrhage, similar to that observed in our infected animals, nearby small arteries and veins where moderate oedema was also found, but without significant cellular infiltration.^29^ In some cases, perivascular haemorrhage can cause death, even if the other organs are not damaged. In this context, it was concluded that brain involvement in naturally acquired human YFV infection is minimal, which is probably related to the general bleeding tendency and, therefore, is not a true encephalitis.^29^ These findings are adequate to explain most of the neurological complications of YF, such as eyelid ptosis and partial facial paralysis.^30^ In rhesus monkeys, YFV is neurotropic administered by any route and results in severe encephalitis that differs radically from the mild encephalitis described in humans.^25^ For an accurate understanding of the inherent mechanisms of injury in the various organs and systems in YFV infection, studies using different techniques are necessary in order to have a global and at the same time detailed view of the physio pathological events and changes observed in the various organs of these animals.

Although there was some variation in the intensity or extent of involvement of tissue damage, it is evident that pathological processes occurring in humans and squirrel monkeys during YFV infection are similar. Thus, the squirrel monkeys (*Saimiri* spp.) is a good model for studies on the pathogenesis of YF.
