# Supplementary material for: Experimental yellow fever virus infection in the squirrel monkey (Saimiri spp.) I: gross anatomical and histopathological findings in organs at necropsy
Source: Mem Inst Oswaldo Cruz. 2020 Nov 9;115:e190501. doi: 10.1590/0074-02760190501 (PMC7651848; doi:10.1590/0074-02760190501)
Supplement: Supplementary file 1 [file 1678-8060-mioc-115-e190501-s.pdf]

TABLE

Main histopathological changes observed in monkeys' squirrels (*Saimiri* spp.) infected with yellow fever virus (YFV)

| Organ | dpi | Histopathological changes |
|---|---|---|
| Heart | 1 and 6 | Myocardium with areas close to the endocardium containing myofibrers with increased eosinophilia, nuclear pyknosis and cytoplasmic vacuoles |
| | 2 | Discrete areas of incipient necrosis in ischemic the endocardium |
| | 3 | Discreet areas of subendocardial incipient ischemic necrosis bordered by vacuolated myocytes |
| Inoculated area | 1 and 2 | Dermis presented mild multifocal perivascular mononuclear infiltrate; inflammatory infiltrate with extensive focal polymorphonuclear and mononuclear cells in the panicle. |
| | 3 | Light focal perivascular mononuclear infiltrate in the adipose panicle |
| | 4 and 5 | Mild perivascular mononuclear infiltrate in the adipose panicle |
| | 6 | Discrete multifocal perivascular mononuclear infiltrate |
| | 7 | Mononuclear infiltrate in the light focal superficial dermis |
| | 10-20 | Light multifocal perivascular mononuclear infiltrate in the adipose panicle and adjacent muscle |
| | 30 | Light multifocal mononuclear infiltrate in the adipose panicle and adjacent areas |
| Liver | 1 | Cytoplasmic swelling and pallor, thin hepatocyte vacuolisation mainly in Z3; moderate diffuse haemosiderosis in Kupffer cells and macrophages in PT and hepatocytes; mononuclear inflammatory infiltrate in mild multifocal PT; haemorrhage in Z2/Z3, steatosis and lytic necrosis in hepatocytes. |
| | 2 | Hepatocytes with mild cytoplasmic pallor at Z3 and mild diffuse haemosiderosis; dilation of sinusoid capillaries at Z3; haemorrhage in Z2/Z3, steatosis and lytic necrosis in hepatocytes. |
| | 3 | Light diffuse swelling and cytoplasmic pallor; mild diffuse haemosiderosis in Kupffer cells; mononuclear inflammatory infiltrate with rare eosinophils in mild multifocal PT; mononuclear inflammatory infiltrate with random multifocal neutrophils between the strands of hepatocytes; haemorrhage in Z2/Z3, steatosis and lytic necrosis in hepatocytes, |
| | 4 | Light to moderate multifocal PT mononuclear inflammatory infiltrate and mononuclear infiltrate with random multifocal neutrophils between the hepatocyte strands; diffuse hepatocyte swelling and cytoplasmic pallor; mild diffuse haemosiderosis; haemorrhage in Z2/Z3, steatosis and lytic necrosis in hepatocytes. |
| | 5 | Mild diffuse haemosiderosis and moderate hepatocyte cytoplasmic vacuolation and PT; individual hepatocyte necrosis is rarely found; haemorrhage in Z2/Z3, steatosis and lytic necrosis in hepatocytes. |
| | 6 | Coagulation necrosis in Z2/Z3 massive and marked diffuse associated with congestion and haemorrhage; hepatocytes with granulated and eosinophilic cytoplasm, karyolysis, pyknosis and karyorexia; Kupffer cells were found at Z2/Z3 with abundant, granulated and eosinophilic cytoplasm, vacuoles and nuclear debris (Councilman bodies); endothelial cells present moderate swelling and occasionally there are clusters of polymorphonuclear cells. In the PT and remaining hepatocytes present swelling and vacuolisation of the cytoplasm; rare intranuclear eosinophilic linear inclusions (including brick or "blochlike") inclusions have been observed in hepatocytes; haemorrhage in Z2/Z3, steatosis and lytic necrosis in hepatocytes. |
| | 7 | Mononuclear infiltrate predominantly in mild multifocal PT and mild diffuse vacuolisation of hepatocytes |
| | 10 | Hepatocyte swelling with diffuse mild to moderate cytoplasmic vacuolation, more intense in Z3; light multifocal mononuclear infiltrate. |
| | 20 | Diffuse hepatocyte vacuolisation, more intense in Z3; mild haemosiderosis. |
| | 30 | Hepatocyte swelling and vacuolisation mainly in mild diffuse Z3; mild haemosiderosis and mild multifocal mononuclear infiltrate. |
| Spleen | 1 | Pyknotic nodules and debris within follicles and in the cytoplasm of follicular macrophages, with mild lymphoid depletion |
| | 2 | Mild lymphoid depletion with discrete neutrophil infiltrate; some with pyknotic nuclei, free debris in the follicles or in macrophages. |
| | 3 | Centrofollicular rarefaction with pyknotic nuclei and debris; prominent and binucleated macrophages, with eosinophilic material and cytoplasmic nuclear remains. |
| | 4 | White pulp with centrofollicular rarefaction, moderate depletion with lymphocytes with pyknotic nuclei and prominent macrophages containing nuclear remains and eosinophilic material in the cytoplasm |
| | 5 | White pulp follicles with retracted lymphocytes with pyknotic nuclei and prominent macrophages containing lumpy eosinophilic material and nuclear remains |
| | 6 | Rare pyknotic lymphocytes and nuclear debris; mild extramedullary haematopoiesis; well-defined follicle; bleeding was also observed in the centre of the cortical region. |
| | 7 | Follicular macrophages containing eosinophilic cell debris and cell debris in the cytoplasm; mild diffuse lymphoid hyperplasia and congestion with necrosis in the follicles that was characterised by the presence of debris in the macrophage cytoplasm. |
| | 10 | Moderate centrofollicular degeneration and presence of macrophages with cellular debris |
| | 20 | Individual necrosis of small groups of lymphocytes with the presence of macrophages containing cellular debris; neutrophils with swollen cytoplasm. |
| | 30 | Rarefaction of follicles with individual necrosis of small groups of lymphocytes, neutrophils and macrophages containing cellular debris |
| Lung | 1-7 | Light diffuse congestion |
| | 6 | Oedema, congestion, severe haemorrhage and mononuclear inflammatory infiltrate |
| | 10 | Mild diffuse congestion with multifocal atelectasis; rare areas of tubular mineralisation in the spinal cord. |
| | 20 | Anthracnose and mild diffuse congestion; rare areas of tubular mineralisation in the spinal cord. |
| | 30 | Light diffuse congestion |
| Kidney | 2-5 | Light multifocal interstitial mononuclear infiltrate and rare eosinophils |
| | 6 | Discrete multifocal interstitial mononuclear infiltrate; cytoplasm vacuolisation, haemorrhagic spots on Bowman's capsule and tubular necrosis. |
| | 7 | Light diffuse congestion with areas of discrete multifocal tubular calcification in the spinal cord |
| | 10-20 | Rare foci of mineralisation in the spinal cord |
| Stomach | 1 and 2 | Light multifocal mononuclear infiltrate with eosinophils on the *lamina propria* |
| | 3 | Occasional individual cells in the mucosa with eosinophilia and pyknosis |
| | 4 | Mononuclear infiltrate with eosinophils in the light diffuse submucosa |
| | 6 | Moderate number of acidophilic cells with nuclear pyknosis and increased eosinophilia |
| | 10 | Light mononuclear infiltrate with eosinophils in the submucosa |
| Axial lymph nodes | 2-6 | Moderate haemosiderosis and non-evident follicles |
| | 7 | Mild diffuse lymphoid hyperplasia and congestion |
| | 10 | Moderate lymphocyte necrosis characterised by the presence of debris in the macrophage cytoplasm |
| | 20 | Mild individual lymphocyte necrosis in the follicles characterised by the presence of debris in the macrophage cytoplasm; there is a small amount of foamy macrophages in the spinal cord and occasional clear vacuoles between the lymphocytes in the cortex. |
| | 30 | Discreet individual lymphocyte necrosis in the follicles with a small amount of foamy macrophages in the spinal cord |
| Brain | 2 | Parietal, occipital and frontal cortex with focal calcification in the leptomeninges extending to the superficial layer of the cortex |
| | 3 | Parietal cortex with mononuclear inflammatory infiltrate in mild multifocal leptomeninges |
| | 6 | Occipital cortex with deep layers of white matter; there is a vacuolisation area of the neuropile where there are astrocytes with abundant eosinophilic cytoplasm; occasionally, in other areas in the white matter without vacuolisation. |
| | 7 | Cerebellum with discrete vacuolisation in white matter |

dpi: days post-infection; PT: portal tract.

cc BY
